# The Effect of N-Acetylcysteine on Behavioral Sensitization to Methamphetamine in Mice

**DOI:** 10.33549/physiolres.935471

**Published:** 2025-04-01

**Authors:** Alena MÁCHALOVÁ, Leoš LANDA, Jan MÁCHAL, Regina DEMLOVÁ, Jiří SLÍVA

**Affiliations:** 1 Department of Pharmacology, Faculty of Medicine, Masaryk University, Brno, Czech Republic; 2 Cannabis Facility, International Clinical Research Centre, St. Anne’s University Hospital, Brno, Czech Republic; 3 Department of Pathophysiology, Faculty of Medicine, Masaryk University, Brno, Czech Republic; 4 Masaryk Memorial Cancer Institute, Brno, Czech Republic; 5 Department of Pharmacology, Third Faculty of Medicine, Charles University, Praha, Czech Republic

**Keywords:** N-acetylcysteine, Methamphetamine, Behavioral sensitization

## Abstract

Behavioral sensitization is a phenomenon occurring after repeated administration of various psychotropic substances and it is characterized by gradually increasing response to the particular drug. It has been described for majority of addictive substances including amphetamines. It is considered to reinstate drug-seeking behaviour and plays important role in the processes associated with drug abuse and addiction. There are published reports, particularly on preclinical level, that N-acetylcysteine (NAC) may affect addictive properties of different classes of drugs (e.g., cocaine, heroin, alcohol, cannabinoids, nicotine). Since the lack of information on possible effects of NAC on amphetamine derivatives we decided to test possible influence of this substance on behavioral sensitization to methamphetamine (MET) in the mouse open field test. Our results have shown a decreased acute stimulatory effect of MET caused by NAC and moreover, there was a non-significant trend of attenuated development of behavioral sensitization to MET after simultaneous long-term administration of MET and NAC. This suppression of MET stimulatory effects therefore suggested on the preclinical level possible promising efficacy of NAC on addictive properties associated with MET similarly as it was demonstrated by other authors in association with cocaine or heroin.

## Introduction

Behavioral sensitization is a relatively new concept that was consistently described in the last decade of the twentieth century [1] and soon become an important target of experimental pharmacology dealing with the research of dependence-producing substances. The most typical feature of behavioral sensitization is a progressive increase in locomotor activity following repeated administration of several drugs [2]. It is also sometimes termed “reverse tolerance” [3] in contrast to “classical” tolerance, which is a phenomenon characterized by the decreasing response of an organism following repeated drug administration. From the point of view of pre-clinical research, the ability to elicit sensitization is fundamental property of drugs with addictive potential, enabling the observation of some of their characteristic features that were hidden in the classical “tolerance-dependence” model. Behavioral sensitization is usually manifested after both repeated doses and an application of a dose administered after a certain withdrawal period (wash-out period).

Sensitization has been observed after repeated administration of various substances, and is well-documented for example in ethanol [4], nicotine [5], caffeine [6], cannabinoids [7], psychostimulants [8–10], and opioids [11]. It has been also reported that an increased response to a drug may be elicited by previous repeated administration of a drug different from the drug tested (i.e., cross-sensitization). This was seen for example in heroin effects after pre-treatment with THC [12] or in amphetamine after repeated treatment with nicotine [13]. It is widely accepted that both behavioral sensitization and cross-sensitization are consequences of substance-induced neuroadaptive changes in a circuit which involves particularly dopaminergic, glutamatergic and GABAergic interconnections between the ventral tegmental area, nucleus accumbens, prefrontal cortex and amygdala [14, 15]. They are considered a useful animal model for determining the neural basis of dependency and their original principles still seem well supported [16,17].

In our previous studies we tested the possible effects of various psychotropic substances on behavioral sensitization to methamphetamine (MET). We investigated the effects of the cannabinoid CB_1_ receptor agonist methanandamide, CB_1_ receptor antagonist AM 251 and CB_2_ receptor agonist JWH 015 [8,9], the effects of the glutamatergic NMDA receptor antagonists felbamate [18] and memantine [19], effect of nootropic piracetam [20] and finally dopamine D_2_ receptor antagonist sertindole [21].

In the present experiment we tested the possible effect of N-aceylcysteine (NAC) on the sensitizing phenomenon associated with repeated administration of MET. NAC (N-acetyl derivative of the natural amino acid L-cysteine) is typically used as a reliable antidote for intoxication with paracetamol (acetaminophen). Overdose with paracetamol leads to the production of more toxic metabolites that deplete the glutathione reserves, which results in their accumulation and hence tissue injury by binding to cellular macromolecules. NAC repletes glutathione reserves by providing cysteine, an essential precursor in glutathione production [22]. Glutathione is an antioxidant and facilitates conjugation to toxic metabolites. NAC is also used as a mucolytic drug (destroying or dissolving mucus) within cough treatment. It hydrolyses the disulfide bonds of mucus proteins thereby decreasing mucus viscosity and facilitating expectoration. Nevertheless, it has been shown, that the mechanism of action of NAC is much more complex and it also involves some central effects. It was particularly described that NAC is involved in regulating the glutamate level *via* glutamate transporter type 1 (responsible for the largest part of neural glutamate transport) and controls glutamate clearance [23].

Numerous studies refer to the important involvement of glutamatergic transmission in the process of behavioral sensitization [24–27] and it has been described that NAC also interferes with processes associated with drug dependency. For example, some reports indicated that the application of NAC restored accumbal glutamate levels and reduced the reinstatement of cocaine-seeking behaviors [23]. Many other examples of possible NAC role in various substance use disorders can be found e.g., in the review by Smaga *et al*. [28].

As mentioned previously we used two glutamatergic drugs (NMDA receptor antagonists felbamate and memantine) in our earlier studies. We tested their possible effects on behavioral sensitization to methamphetamine [18,19]. Since the suggested interference of NAC with the glutamatergic system and to extend our research, we, therefore, focused in the present study on possible changes in the sensitization to methamphetamine stimulatory effects after NAC administration in mice.

## Materials and methods

### Animals

Male mice, n=104, (strain ICR, TOP-VELAZ s.r.o., Prague, Czech Republic) with an initial weight of 18–21g were used and housed with free access to water and food in a room with controlled humidity and temperature, that was maintained under a 12-h phase lighting cycle. In order to minimise possible variability due to circadian rhythms the behavioral observations were always performed in the same period between 1:00 p.m. and 3:00 p.m.

### Apparatus

Locomotor activity was recorded using an open-field equipped with Actitrack (Panlab, S. L., Spain). This device consists of two square-shaped frames that deliver beams of infrared rays into the space inside the square. A plastic box in this square acts as an open-field arena (base 30 x 30 cm, height 20 cm) where the animal can move freely. The apparatus software measures and evaluates the locomotor activity of the animal by registering the beam interruptions caused by movements of the body. Using this equipment, we determined the trajectory in cm per 4 minutes (Distance Travelled).

### Drugs

Vehicle and all drugs were always given in a volume adequate to drug solutions (10 ml/kg).

(+)Methamphetamine (d-N,α-Dimethylphenyl-ethylamine;d-Desoxyephedrine) (Sigma Chemical Co.) dissolved in saline.

N-acetylcysteine was purchased from a pharmacy as a commercially available solution and dissolved in saline.

All drug doses were adjusted based on literature data and results from our earlier behavioral experiments.

### Procedure

Before the experiment started, animals were given 7 days to acclimate to the housing environment. At the beginning of the experiment (Day 1), animals were randomly divided into eight groups (n_=_13 per group) and tested in the open-field to check proper randomization. On Days 3–9, animals were treated daily as follows: a) n_1,2_: saline (SAL) at the doses of 10 ml/kg/day; b) n_3,4_: N-acetylcysteine (NAC) at the doses 50 mg/kg/day; c) n_5,6_: methamphetamine (MET) at the doses of 2.5 mg/kg/day; d) n_7,8_: NAC + MET at the doses of 50 mg/kg/day and 2.5 mg/kg/day, respectively. On Day 10 all animals were administered the first challenge dose of MET (2.5 mg/kg). The administration during Days 11 – 17 were as follows: n_1_: SAL at the doses of 10 ml/kg/day; n_2_: MET at the doses of 2.5 mg/kg/day; n_3_: NAC at the doses 50 mg/kg/day; n_4_: MET at the doses of 2.5 mg/kg/day; n_5_: NAC at the doses 50 mg/kg/day; n_6_: SAL at the doses of 10 ml/kg/day; n_7_: NAC at the doses 50 mg/kg/day; n_8_: SAL at the doses of 10 ml/kg/day. On Day 18, the second challenge dose of MET was given, and on Day 24 the third challenge dose of MET was administered (2.5 mg/kg) to all animals. For graphic illustration see Table 1.

All substances were administered intraperitoneally; MET was applied 30 minutes before testing, and NAC was applied 120 minutes before testing. Changes in Distance Travelled (horizontal locomotion) were measured for a period of 4 minutes in the open field on Day 3 to evaluate drugs’ acute effects and on Days 10, 18 and 24 to evaluate the sensitizing phenomenon.

The experimental protocol of the experiment complies with the European Community guidelines for the use of experimental animals and was approved by the Animal Care Committee of the Masaryk University Brno, Czech Republic.

### Statistical methods

To compare the data at the baseline, following the acute dose and after the first challenge by MET, one-way analysis of variance (ANOVA) with Tukey *post hoc* test was employed to compare distance among four groups pre-treated by: (1, 2) SAL, (3, 4) NAC, (5, 6) MET, or (7, 8) by combination of NAC + MET (i.e. COMB group). After the first challenge by methamphetamine, the mice started to receive the second drug, and thus eight groups were defined: (1) SAL/SAL, (2) SAL/MET, (3) NAC/NAC, (4) NAC/MET, (5) MET/NAC, (6) MET/SAL, (7) COMB/MET and (8) COMB/SAL. The distance in these eight groups was then compared by one-way ANOVA with Tukey *post hoc* test similarly to the previous cases after two or three challenge doses. To identify possible baseline differences, the eight groups were compared at the baseline as well. Normality of the data was checked by Kolmogorov-Smirnov test and normal probability plots. Differences with p<0.05 were considered to be statistically significant; p-values refer to adjusted values in case of multiple comparison following ANOVA. The dataset was searched for outlying values based on descriptive statistics. There was one outlying value in the NAC during the control measurement with a z-score of 3.16 that, however, is expected given the size of the dataset and did not violate the assumption of normal distribution; therefore, it was included in the data analysis. All analyses were performed by Statistica, ver. 14.0.0.15, TIBCO Software.

## Results

**Fig. 1:**
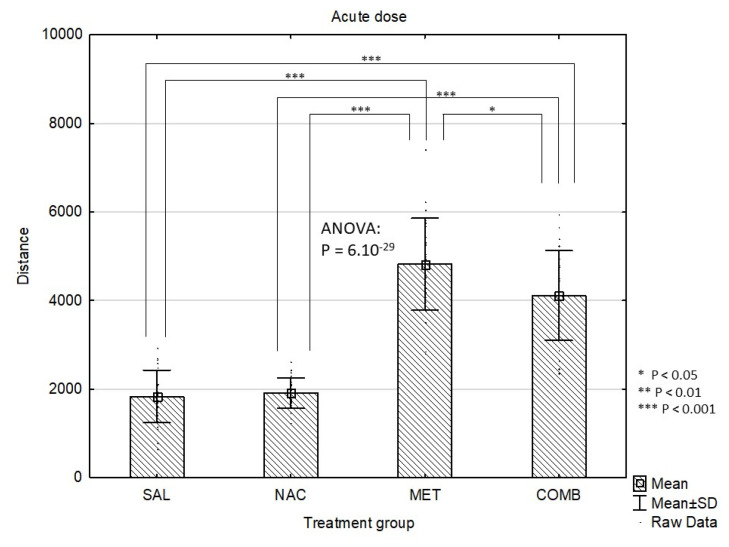
Effects of drug treatments (acute dose) on Distance Travelled (cm/4 min) in the mouse open field test shown as mean values ± SD. SAL=saline, NAC=N-acetylcysteine, MET=methamphetamine, COMB=NAC+MET

**Fig. 2:**
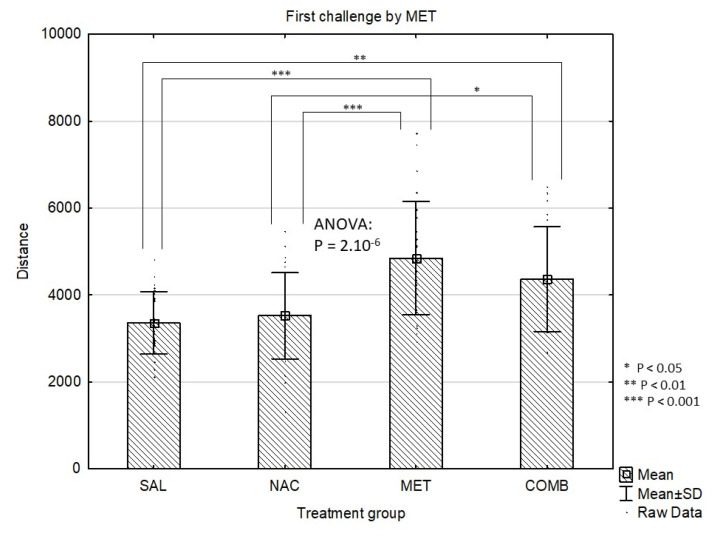
Effects of drug treatments (first challenge dose) on Distance Travelled (cm/4 min) in the mouse open field test shown as mean values ± SD. SAL=saline, NAC=N-acetylcysteine, MET=methamphetamine, COMB=NAC+MET

**Fig. 3:**
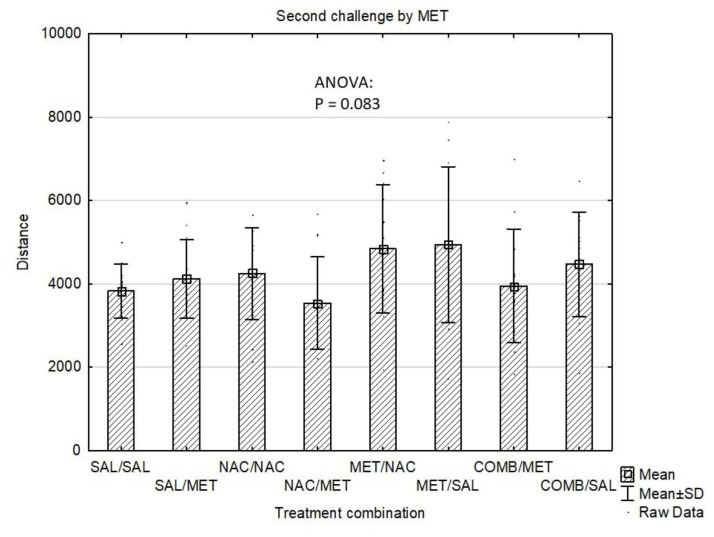
Effects of drug treatments (second challenge dose) on Distance Travelled (cm/4 min) in the mouse open field test shown as mean values ± SD. SAL=saline, NAC=N-acetylcysteine, MET=methamphetamine, COMB=NAC+MET

**Fig. 4:**
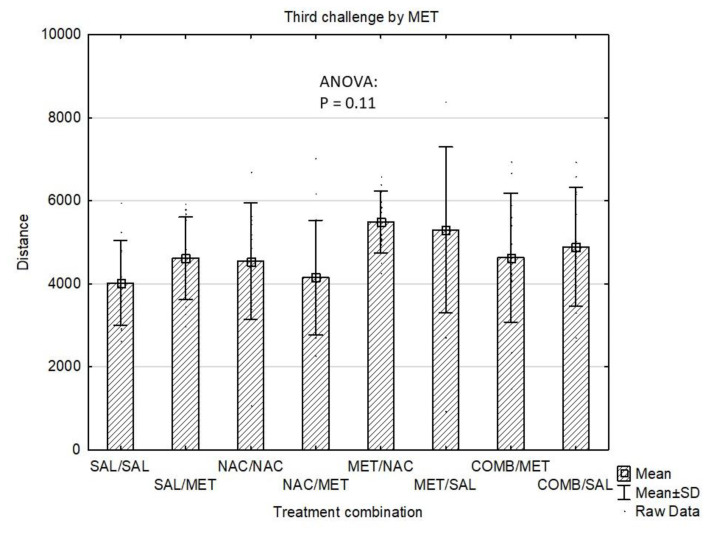
Effects of drug treatments (third challenge dose) on Distance Travelled (cm/4 min) in the mouse open field test shown as mean values ± SD. SAL=saline, NAC=N-acetylcysteine, MET=methamphetamine, COMB=NAC+MET

In whole study group, the Distance Travelled at the baseline (mean±standard deviation) was 2258±529 and did not differ among groups according to the first pre-treatment (4 groups, p=0.31) or according to the treatment combination (8 groups, p=0.78). Throughout the experiment, the Distance Travelled gradually increased (acute dose: 3168±1546; first challenge: 4021±1226; second challenge: 4235±1312; third challenge: 4695±1407; p<10-4). After the acute dose, significant differences were observed between the four groups (p=6.10–29). Specifically, MET group had higher Distance Travelled compared to SAL (p=10-4) and NAC (p=10-4). Similarly, in COMB group, the Distance Travelled was also higher than in both SAL (p=10-4) and NAC (p=10-4). The COMB group had lower Distance Travelled compared to MET, p=0.011) (Fig. 1). Some of the differences also remained significant after the first challenge dose of MET (overall p=2.10–6). After the first challenge, MET group still had higher Distance Travelled compared to both SAL (p=2.10–4) and NAC (p=2.10–4), similarly to COMB group compared to SAL (p=6.10-3) and NAC (p=0.029) (Fig. 2). After the first challenge, the study groups were further split according to the treatment combination, making the total of 8 groups. These eight groups also did not differ according to the baseline distance (p=0.78). Following further challenges by methamphetamine, the Distances Travelled after the second (p=0.083) and third (p=0.11) challenge were not significantly different between the eight groups (Fig. 3 and 4).

## Discussion

NAC is a commonly prescribed or even “over-the-counter” available substance used as a mucolytic and antioxidant agent. What is more, NAC has been recently proposed as a drug alleviating the neurobiological consequences of the addictive substances misuse. The disbalance of glutamate and dopamine homeostasis in CNS following the application of drugs of abuse is notorious and has been widely discussed [29]. The proposed NAC mechanisms of action in addiction are based on influencing glutamate, the most important excitatory neurotransmitter in the mammal brain. The action of NAC on dopamine levels seems to be indirect and results from changes in glutamate signalization.

Two molecular transporters are essential for maintaining glutamate extracellular levels upon its release: glutamate transporter 1 (GLT-1) and cystine-glutamate antiporter (x_c_^−^). Both these transporters were shown to be influenced by exposure to addictive substances and NAC to have the potency to modulate these changes. GLT-1 is a major brain transporter system, and it allows the co-transportation of glutamate with three Na^+^ and one H^+^ into the cells in exchange for one K^+^. Its proper function provides a major portion of glutamate uptake and is crucial for life as it prevents the spillover of glutamate from the synaptic cleft to surrounding extracellular space. Changes in GLT-1 expression following the administration of drugs of abuse are different in distinct brain regions and drug classes and probably also depend on the dosing regimen. Still, generally, it is recognized that after long-term usage, there is a down-regulation of GLT-1. NAC, as a GLT-1 modulating drug, can restore glutamate uptake *via* GLT-1, so its effects may be at least partially explained by this activity [30].

Cystine-glutamate antiporter (x_c_^−^) allows the exchange of anionic forms of cystine and glutamate in a 1:1 ratio. Due to physiologically low cystine concentration in the cytoplasm the import of cystine and glutamate export prevails [31]. NAC is a prodrug, which, after metabolization, serves as a source of cystine, which in turn activates the x_c_^−^ transporter to exchange extracellular cystine for glutamate. This mechanism may lead to the normalisation of extracellular glutamate levels. Proper levels of glutamate are responsible for maintaining tonic activation of inhibitory mGlu2/3 receptors and modulation of signalling between the prefrontal cortex and nucleus accumbens, brain regions heavily involved in the process of addiction [32].

The antioxidant and neuroprotective effects of NAC are also important. Administration of different drugs can disturb redox balance and lead to oxidative stress. NAC as a potent antioxidant and glutathione precursor can prevent or reverse such changes [33].

There are published reports, both preclinical and clinical, concerning NAC efficacy in treatment of addiction to different classes of substances [28]. While NAC showed results in preclinical models of addiction to cocaine, heroin, alcohol, cannabinoids and nicotine, very few works concerned addiction to amphetamine or its derivatives. There is also a lack of studies testing NAC properties in a broader variety of animal species. In our experimental design, we have tested its efficacy in a mouse model of behavioral sensitization to methamphetamine. According to our knowledge, to date, no such study has been performed before.

The most important finding in our experiment is, that NAC at the dose of 50 mg/kg decreased the acute psychostimulant effect of MET (2.5 mg/kg). Our results also suggested attenuation of the development of behavioral sensitization by simultaneous long-term application of NAC with MET. Although this effect was not statistically significant in our settings, it may fully appear in different paradigms, as was shown in rats [34] when the dose of 100 mg/kg NAC administered in combination with MET (2 mg/kg) for 5 consecutive days effectively prevented the development of behavioral sensitization to MET. The acute effects of MET (2 mg/kg) were suppressed by pre-treatment with NAC in a dose-dependent manner; the effect was statistically significant at the dose of 300 mg/kg NAC in this study [34]. NAC pre-treatment (10 or 30 mg/kg) also attenuated MET-induced increase in the body temperature as well as the level of dopamine in rat striatum (induced by 4 doses of MET 7.5 mg/kg x 4) [34]. These results of NAC acute doses obtained in rats are in agreement with our findings in mice.

In the female rat self-administration model, on the other hand, NAC (30, 60 or 120 mg/kg) administered prior to self-administration or extinction sessions had no significant effect on responding to MET (0.05 kg/ kg/infusion) or MET-triggered (0.25 mg/kg) reinstatement of extinguished behavior [35].

These results are contradicted by a similar experiment when following MET self-administration (20 μg/bolus), male rats received either NAC (100 mg/kg) or saline during extinction prior to extinction sessions. Authors demonstrated that chronic NAC treatment inhibited cued MET seeking. They also showed that MET exposure had no effect on GLT-1 expression or glutamate clearance in the core of nucleus accumbens, or on certain cytoskeletal structures in astrocytes. This finding differs from the effects of cocaine administrations which are much better described in the literature. Authors hypothesize that the effect of NAC may involve additional molecular mechanisms in the case of MET use compared to cocaine, such as the previously mentioned x_c_^−^ transporter, *via* which NAC may restore basal glutamate levels in nucleus accumbens core [36].

Although preclinical studies are showing promise, the results of clinical studies are somewhat mixed, probably due to lower compliance of participants and the possible confoundment of experimental conditions in their everyday lives compared to standardized laboratory environments.

In a double-blind, placebo-controlled clinical trial, 32 MET-dependent participants received either NAC (1200 mg/day) or placebo for 4 weeks, followed by crossover intervention for another 4 weeks. The results showed significantly reduced cravings during NAC treatment compared to the placebo. In the group treated first with NAC, craving scores were increased after crossover to placebo [37].

On the contrary, another double-blind, placebo-controlled trial in humans demonstrated unsatisfactory NAC effects in treating MET dependence. Participants (n=153) with a history of MET use received orally either NAC (2400 mg/day) or placebo. There was no effect of NAC on craving, MET intake or any behavioral parameters or psychiatric symptoms assessed in the study when compared to placebo. Authors, however, discuss poor adherence to the treatment and, possibly, too low an NAC dose [38].

Our results have convincingly demonstrated a decrease in acute effect of methamphetamine caused by N-acetylcysteine and moreover there was a trend (yet not significant) of attenuated development of behavioral sensitization to methamphetamine following simultaneous long-term administration of methampheta-mine and N-acetylcysteine. Further research would be required using different paradigms and doses, nevertheless our findings suggested on the preclinical level possible promising efficacy of NAC in treatment of addictive disorders associated with amphetamine derivatives similarly as it was demonstrated by other authors for example with respect to cocaine or heroin.

## Figures and Tables

**Table 1:** Experimental design

Days	1	3	4 – 9	10	11–17	18	24

Actitrack	↓	↓		↓		↓	↓

Group	Control of randomisation	Acute dose	Applications	1st challenge	Applications	2nd challenge	3rd challenge
1	no	saline	saline	MET	saline	MET	MET
2	no	saline	saline	MET	MET	MET	MET
3	no	NAC	NAC	MET	NAC	MET	MET
4	no	NAC	NAC	MET	MET	MET	MET
5	no	MET	MET	MET	NAC	MET	MET
6	no	MET	MET	MET	saline	MET	MET
7	no	NAC + MET	NAC + MET	MET	NAC	MET	MET
8	no	NAC + MET	NAC + MET	MET	saline	MET	MET

NAC = N-acetylcysteine, MET = methamphetamine

## References

[1] Robinson TE, Berridge KC (1993). The neural basis of drug craving: an incentive-sensitization theory of addiction. Brain Res Brain Res Rev.

[2] Nona CN, Nobrega JN (2018). A role for nucleus accumbens glutamate in the expression but not the induction of behavioural sensitization to ethanol. Behav Brain Res.

[3] Demontis F, Falconi M, Canu D, Serra G (2015). Memantine prevents “bipolar-like” behavior induced by chronic treatment with imipramine in rats. Eur J Pharmacol.

[4] Xu S, Kang UG (2017). Characteristics of ethanol-induced behavioral sensitization in rats: Molecular mediators and cross-sensitization between ethanol and cocaine. Pharmacol Biochem Behav.

[5] Thompson MF, Poirier GL, Dávila-García MI, Huang W, Tam K, Robidoux M, Dubuke ML, Shaffer SA, Colon-Perez L, Febo M, DiFranza JR, King JA (2018). Menthol enhances nicotine-induced locomotor sensitization and in vivo functional connectivity in adolescence. J Psychopharmacol.

[6] Kumar S, Verma L, Jain NS (2018). Role of histamine H1 receptor in caffeine induced locomotor sensitization. Neurosci Lett.

[7] Cadoni C, Valentini V, Di Chiara G (2008). Behavioral sensitization to delta 9-tetrahydrocannabinol and cross-sensitization with morphine: differential changes in accumbal shell and core dopamine transmission. J Neurochem.

[8] Landa L, Sulcova A, Slais K (2006a). Involvement of cannabinoid CB1 and CB2 receptor activity in the development of behavioural sensitization to methamphetamine effects in mice. Neuro Endocrinol Lett.

[9] Landa L, Slais K, Sulcova A (2006b). Impact of cannabinoid receptor ligands on behavioural sensitization to antiaggressive methamphetamine effects in the model of mouse agonistic behaviour. Neuro Endocrinol Lett.

[10] Kang BJ, Song SS, Wen L, Hong KP, Augustine GJ, Baik JH (2017). Effect of optogenetic manipulation of accumbal medium spiny neurons expressing dopamine D2 receptors in cocaine-induced behavioral sensitization. Eur J Neurosci.

[11] Perreau-Lenz S, Hoelters LS, Leixner S, Sanchis-Segura C, Hansson A, Bilbao A, Spanagel R (2017). mPer1 promotes morphine-induced locomotor sensitization and conditioned place preference via histone deacetylase activity. Psychopharmacology (Berl).

[12] Singh ME, McGregor IS, Mallet PE (2005). Repeated exposure to Delta(9)-tetrahydrocannabinol alters heroin-induced locomotor sensitisation and Fos-immunoreactivity. Neuropharmacology.

[13] Adams E, Klug J, Quast M, Stairs DJ (2013). Effects of environmental enrichment on nicotine-induced sensitization and cross-sensitization to d-amphetamine in rats. Drug Alcohol Depend.

[14] Vanderschuren LJ, Kalivas PW (2000). Alterations in dopaminergic and glutamatergic transmission in the induction and expression of behavioral sensitization: a critical review of preclinical studies. Psychopharmacology (Berl).

[15] Nestler EJ (2001). Molecular basis of long-term plasticity underlying addiction. Nat Rev Neurosci.

[16] Steketee JD, Kalivas PW (2011). Drug wanting: behavioral sensitization and relapse to drug-seeking behavior. Pharmacol Rev.

[17] Berridge KC, Robinson TE (2016). Liking, wanting, and the incentive-sensitization theory of addiction. Am Psychol.

[18] Landa L, Slais K, Sulcova A (2012a). The effect of felbamate on behavioural sensitization to methamphetamine in mice. Vet Med (Praha).

[19] Landa L, Slais K, Sulcova A (2012b). The effect of memantine on behavioural sensitization to methamphetamine in mice. Vet Med (Praha).

[20] Machalova A, Slais K, Vrskova D, Sulcova A (2011). Enhancement of psychostimulant effect and development of behavioural sensitisation to methamphetamine in mice by combined treatment with piracetam. Act Nerv Super Rediviva,.

[21] Landa L, Slais K, Sulcova A (2012). The effect of sertindole on behavioural sensitisation to methamphetamine in mice. Vet Med (Praha).

[22] Ershad M, Naji A, Vearrier D (2023). N-Acetylcysteine. StatPearls [Internet].

[23] Reissner KJ, Gipson CD, Tran PK, Knackstedt LA, Scofield MD, Kalivas PW (2015). Glutamate transporter GLT-1 mediates N-acetylcysteine inhibition of cocaine reinstatement. Addict Biol.

[24] Ohmori T, Abekawa T, Muraki A, Koyama T (1994). Competitive and noncompetitive NMDA antagonists block sensitization to methamphetamine. Pharmacol Biochem Behav.

[25] Subramaniam S, Rho JM, Penix L, Donevan SD, Fielding RP, Rogawski MA (1995). Felbamate block of the N-methyl-D-aspartate receptor. J Pharmacol Exp Ther.

[26] Tzschentke TM, Schmidt WJ (2003). Glutamatergic mechanisms in addiction. Mol Psychiatry.

[27] Lee KW, Kim HC, Lee SY, Jang CG (2011). Methamphetamine-sensitized mice are accompanied by memory impairment and reduction of N-methyl-d-aspartate receptor ligand binding in the prefrontal cortex and hippocampus. Neuroscience.

[28] Smaga I, Frankowska M, Filip M (2021). N-acetylcysteine in substance use disorder: a lesson from preclinical and clinical research. Pharmacol Rep.

[29] Cheron J, Kerchove d’Exaerde Ad (2021). Drug addiction: from bench to bedside. Transl Psychiatry.

[30] Spencer S, Kalivas PW (2017). Glutamate transport: A new bench to bedside mechanism for treating drug buse. Int J Neuropsychopharmacol.

[31] Bannai S (1986). Exchange of cystine and glutamate across plasma membrane of human fibroblasts. J Biol Chemistry.

[32] Baker DA, Xi ZX, Shen H, Swanson CJ, Kalivas PW (2002). The origin and neuronal function of in vivo nonsynaptic glutamate. J Neurosci.

[33] Möller M, Du Preez JL, Viljoen FP, Berk M, Emsley R, Harvey BH (2013). Social isolation rearing induces mitochondrial, immunological, neurochemical and behavioural deficits in rats, and is reversed by clozapine or N-acetyl cysteine. Brain Behav Immun.

[34] Fukami G, Hashimoto K, Koike K, Okamura N, Shimizu E, Iyo M (2004). Effect of antioxidant N-acetyl-L-cysteine on behavioral changes and neurotoxicity in rats after administration of methamphetamine. Brain Res.

[35] Charntikov S, Pittenger ST, Pudiak CM, Bevins RA (2018). The effect of N-acetylcysteine or bupropion on methamphetamine self-administration and methamphetamine-triggered reinstatement of female rats. Neuropharmacology.

[36] Siemsen BM, Reichel CM, Leong KC, Garcia-Keller C, Gipson CD, Spencer S, McFaddin JA, Hooker KN, Kalivas PW, Scofield MD (2019). Effects of methamphetamine self-administration and extinction on astrocyte structure and function in the nucleus accumbens core. Neuroscience.

[37] Mousavi SG, Sharbafchi MR, Salehi M, Peykanpour M, Karimian Sichani N, Maracy M (2015). The efficacy of N-acetylcysteine in the treatment of methamphetamine dependence: a double-blind controlled, crossover study. Arch Iran Med.

[38] McKetin R, Dean OM, Turner A, Kelly PJ, Quinn B, Lubman DI, Dietze P, Carter G, Higgs P, Sinclair B, Reid D, Baker AL, Manning V, Pas NT, Thomas T, Bathish R, Raftery DK, Wrobel A, Saunders L, Arunogiri S, Cordaro F, Hill H, Hall S, Clare PJ, Mohebbi M, Berk M (2021). N-acetylcysteine (NAC) for methamphetamine dependence: A randomised controlled trial. EClinicalMedicine.

